# Diabetes in childhood cancer survivors: emerging concepts in pathophysiology and future directions

**DOI:** 10.3389/fmed.2023.1206071

**Published:** 2023-08-22

**Authors:** Rusha Bhandari, Saro H. Armenian, Shana McCormack, Rama Natarajan, Sogol Mostoufi-Moab

**Affiliations:** ^1^Department of Pediatrics, City of Hope, Duarte, CA, United States; ^2^Department of Population Sciences, City of Hope, Duarte, CA, United States; ^3^Department of Pediatrics, Children’s Hospital of Philadelphia, Philadelphia, PA, United States; ^4^Department of Diabetes Complications and Metabolism, Arthur Riggs Diabetes and Metabolism Research Institute, City of Hope, Duarte, CA, United States

**Keywords:** diabetes, childhood cancer survivor, insulin resistance, radiation, body composition

## Abstract

With advancements in cancer treatment and supportive care, there is a growing population of childhood cancer survivors who experience a substantial burden of comorbidities related to having received cancer treatment at a young age. Despite an overall reduction in the incidence of most chronic health conditions in childhood cancer survivors over the past several decades, the cumulative incidence of certain late effects, in particular diabetes mellitus (DM), has increased. The implications are significant, because DM is a key risk factor for cardiovascular disease, a leading cause of premature death in childhood cancer survivors. The underlying pathophysiology of DM in cancer survivors is multifactorial. DM develops at younger ages in survivors compared to controls, which may reflect an “accelerated aging” phenotype in these individuals. The treatment-related exposures (i.e., chemotherapy, radiation) that increase risk for DM in childhood cancer survivors may be more than additive with established DM risk factors (e.g., older age, obesity, race, and ethnicity). Emerging research also points to parallels in cellular processes implicated in aging- and cancer treatment-related DM. Still, there remains marked inter-individual variability regarding risk of DM that is not explained by demographic and therapeutic risk factors alone. Recent studies have highlighted the role of germline genetic risk factors and epigenetic modifications that are associated with risk of DM in both the general and oncology populations. This review summarizes our current understanding of recognized risk factors for DM in childhood cancer survivors to help inform targeted approaches for disease screening, prevention, and treatment. Furthermore, it highlights the existing scientific gaps in understanding the relative contributions of individual therapeutic exposures and the mechanisms by which they exert their effects that uniquely predispose this population to DM following cancer treatment.

## Background

For most common pediatric malignancies, five-year survival rates exceed 90% (1); as a result, currently, there are an estimated 500,000 childhood cancer survivors (CCS) in the U.S. alone (2). Despite this success, the growing population of long-term CCS faces a significantly worse quality of health compared to the non-oncology population due to cancer diagnosis and treatment exposures during key developmental periods. Approximately 20 years after cancer diagnosis, more than 25% of CCS have a chronic health condition (e.g., musculoskeletal deficits, cardiovascular disease, endocrinopathies) (3, 4). While advances in treatment and supportive care approaches have reduced the overall burden over time, the risk for specific chronic health conditions such as diabetes mellitus (DM) has increased (3). Among 5-year survivors, the cumulative incidence of self-reported severe, life-threatening, or fatal DM 15 years after primary cancer diagnosis more than doubled in patients diagnosed in the 1990s compared to the 1970s (3). Similar to the general population, in addition to producing immediate adverse health outcomes, DM is also a critical risk factor for subsequent cardiovascular disease, which is a leading cause of death in CCS (5, 6). Compared to the general population, CCS are seven times more likely to die from cardiovascular disease and related morbidities, largely due to treatment exposures such as anthracycline chemotherapy and chest radiation (7). Survivors of adult-onset cancer with DM have a higher risk for cardiovascular disease than do their non-oncology counterparts with DM (8) Thus, prevention and timely management of DM not only leads to a decrease in indirect complications of DM, but also has the potential opportunity to mitigate premature mortality in CCS related to cardiovascular disease.

In addition to premature mortality, the financial burden from DM is substantial. The approximate cumulative cost of medical expenditure, missed work, and decreased productivity from DM in all individuals (i.e., oncology and non-oncology populations) is estimated at 300 billion dollars annually (9). Individuals with DM experience excess financial hardship, financial distress, cost-related medication nonadherence, and food insecurity (10). As CCS have a higher burden of chronic health conditions and spend more of their income on medical care compared to siblings (3, 11), they are particularly vulnerable to the burden of financial toxicity associated with late effects and conditions such as DM.

DM typically develops through progression from normal glucose regulation, to glucose intolerance (prediabetes), and ultimately to glucose dysregulation or overt DM. Each year, approximately 10% of the general population with recognized prediabetes progresses to DM; in contrast, those with prediabetes or insulin resistance who achieve reversion to normal glucose regulation/homeostasis through intervention demonstrate a 56% reduced risk of progression to DM (12). Therefore, early diagnosis and intervention during the prediabetic phase represent important potential opportunities to reduce progression to diabetes. Survivors of childhood cancer have unique risk factors leading to the development of DM. Different mechanistic pathways may be implicated depending on their individual treatment exposures. In this review, we summarize the current understanding of recognized risk factors for DM in CCS to help inform targeted approaches for disease screening, prevention, and treatment. Additionally, this review highlights the existing scientific gaps in understanding the relative contributions of individual therapeutic exposures and the mechanisms by which they exert their effects that uniquely predispose this population to DM following cancer treatment.

## General risk factors

Prediabetes is characterized by insulin resistance and DM is characterized by hyperglycemia due to reduced insulin action and/or relative deficiency in pancreatic insulin secretion with or without decreased insulin sensitivity in target organs. In the general population, obesity coupled with insulin resistance is the strongest risk factor for developing DM; individuals with obesity have an approximately 7-fold risk of developing DM compared to those who are normal weight (13, 14). Other identified risk factors include decreased physical activity and race/ethnicity (Hispanic, Native American, Black) (14).

### Role of body composition

The inverse relationship between body mass index (BMI) and age at DM diagnosis is well recognized (15). Although BMI is a readily available indirect measure of obesity, it fails to account for age- and sex-specific differences in body composition and amounts of specific fat depots such as visceral vs. subcutaneous adipose tissue. Studies assessing body composition in CCS have shown an increased risk for DM independent of BMI (16–18). The increased visceral adiposity seen in survivors is strongly associated with metabolic abnormalities, including insulin resistance, inflammation, and adipokine-associated inflammation, likely compounding the existent risk for DM associated with obesity (18). Visceral adipose tissue (VAT) is the immediate storage site for diet-derived fat. It exhibits high lipid turnover and is the primary source of free fatty acid (FFA) release after an overnight fast (19). Most FFA delivered to the liver originates from visceral fat, particularly in individuals with more VAT, and this effect appears to be greater in women than in men (20, 21).

Sarcopenic obesity, characterized by the concurrent loss of skeletal muscle mass and increase in fat mass, specifically VAT, is an important late effect of cancer treatment that is associated with decreased insulin sensitivity (22–24). Excess adiposity leads to an imbalance in the production of adipocytokines, including an increase in pro-inflammatory cytokines and decrease in adiponectin which results in worsening dyslipidemia and insulin resistance (25). In individuals with obesity, adipocytes become resistant to insulin-stimulated inhibition of lipolysis. This results in increased FFA flux, inflammation, and lipid deposition in skeletal muscle and liver tissue (25). Skeletal muscle is responsible for a majority of the body’s insulin-stimulated glucose disposal, thus this lipid deposition negatively affects glucose uptake in peripheral tissues (25). Importantly, pediatric allogeneic hematopoietic cell transplantation (HCT) survivors have been found to have significantly higher fat mass, higher VAT and subcutaneous adipose tissue, and significantly lower muscle density and lean mass compared to a robust, healthy age-, sex-, and race-matched reference population. These body composition differences were present despite no significant differences in BMI Z-scores between HCT survivors and the reference population (17, 18). The body composition abnormalities further highlight the failure of BMI as a measure to adequately capture discrete disease effects on lean mass and fat mass in HCT survivors. Furthermore, similar body composition abnormalities with normal BMI have also been reported in a longitudinal cohort of adult survivors of allogeneic HCT (26). The observed alterations in body composition likely contribute to treatment-related morbidity and mortality associated with premature atherosclerotic cardiovascular disease, metabolic syndrome, and poor bone health.

Normal insulin signaling typically suppresses hepatic glucose production (27). However, hepatic insulin signaling becomes impaired due to accumulated triglyceride and FFA levels in the liver, leading to increased hepatic gluconeogenesis (15). Excess hepatic glucose production, together with impaired skeletal muscle glucose uptake, further exacerbates hyperglycemia (28). Additionally, FFAs stimulate hepatic inflammatory and pro-thrombotic states, characterized by endothelial activation, which increase cardiovascular risk (29, 30). Nutritional imbalance (e.g., excess fat and carbohydrate consumption relative to needs) can induce an increase in reactive oxygen species (ROS) burden and oxidative stress. Excess ROS in turn lead to increased inflammation and additional downstream effects including mitochondrial dysfunction and epigenetic changes which can persist even after restoration of normoglycemia (15, 31, 32).

Among individuals with insulin resistance, increased lipid deposition within muscle cells (intramyocellular lipids, IMCL) is associated with poor fatty acid oxidation, and decreased insulin sensitivity, independent of BMI (33–35). In oncology patients undergoing potentially curative surgery, higher IMCL is positively correlated with weight loss from pre-illness weight (36). Furthermore, a study of pediatric HCT survivors demonstrated higher fat infiltration of muscle compared to matched controls (18). The location of IMCL also affects insulin sensitivity. A study investigating differences in lipid deposition between endurance-trained athletes and individuals with DM who had similar IMCL levels found that those with DM store lipids in larger droplets in type II muscle fibers in the subsarcolemmal region, where the IMCL can potentially interfere with insulin signaling. In contrast, the trained athletes stored IMCL in smaller lipid droplets in type I muscle fibers in the intermyofibrillar space where they can subsequently be utilized to fuel oxidation (37). This evidence suggests that, in addition to the increased VAT generally seen with sarcopenic obesity, the imbalance of energy intake vs. expenditure and lipid distribution in adipose and muscle depots are potentially important drivers for the development of DM that require further characterization in CCS. Furthermore, the prevalence of adverse body composition, its relative contribution to the development of DM in the context of cancer treatment exposures, and how this risk compares to the general population, have not been well-defined.

## Treatment-related risk factors and increased risk for diabetes mellitus

### Radiation

**Figure 1:**
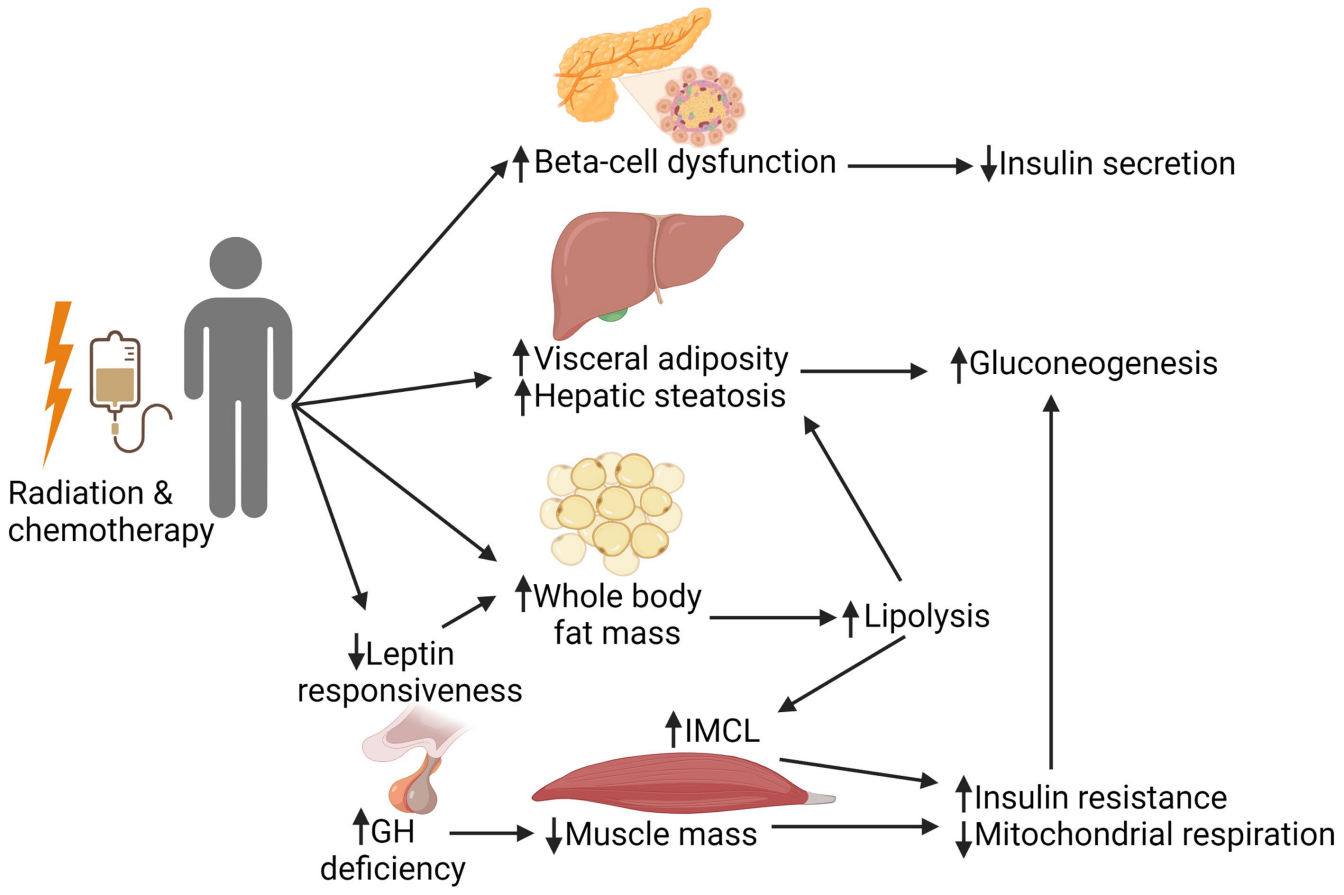
Mechanistic pathways involved in the development of insulin resistance in cancer survivors. Radiation and chemotherapy can have a multitude of complex, interrelated effects on the pancreas, liver, pituitary, and adipose and muscle tissues. Direct effects to the pancreas can lead to beta-cell dysfunction, subsequently resulting in decreased insulin secretion. Direct and indirect effects of treatment negatively affect muscle health (reduced muscle mass and mitochondrial respiration), which is compounded by the fatty infiltration of muscle that is seen with an increase in whole body fat mass. Together, these changes worsen peripheral tissue insulin resistance, leading to increased gluconeogenesis. This creates a cycle of hyperglycemia and decreased insulin sensitivity, with or without adequate insulin production. GH, growth hormone; IMCL, intramyocellular lipids. Created with BioRender.com.

As shown in Figure 1, there are multiple complex and interrelated treatment-associated mechanisms underlying the development of DM in this patient population. This includes effects of radiation and chemotherapy on the pancreas, liver, pituitary, and adipose and muscle tissues, ultimately resulting in a cycle of hyperglycemia and insulin resistance with or without impaired insulin production. Radiation is the strongest treatment-related risk factor associated with the development of subsequent DM in CCS. While cancer treatment has changed across eras, with directed effort to reduce radiation exposure for certain cancers (e.g., chest radiation in patients with Hodgkin lymphoma), radiation remains integral in the upfront treatment for many childhood malignancies such as Wilms tumor, high-risk neuroblastoma, Ewing sarcoma, and a majority of CNS tumors. Total body irradiation (TBI) is still a key component of many conditioning regimens prior to HCT, particularly in patients with relapsed acute lymphoblastic leukemia (ALL) (38, 39).

Specifically, TBI and abdominal radiation are the two most recognized radiation treatment exposures in survivors that subsequently lead to DM. In a study of over 8,500 survivors, after adjusting for relevant clinical and treatment-related factors, survivors treated with TBI demonstrated a 7-fold increased risk (OR 7.2, 95% CI 3.4–15.0) and those with abdominal radiation, a 3-fold increased risk (OR 2.7, 95% CI 1.9–3.8) of DM compared to survivors without these respective exposures (16). Compared to siblings, and after adjusting for BMI, survivors with TBI, abdominal radiation, and cranial radiation were 12.6, 3.4, and 1.6 times as likely to develop DM, respectively (16). The increased risk of DM with TBI exposure is multifactorial, and includes direct effects on pancreatic and adipose tissues as well as the hypothalamic–pituitary axis (40). Childhood cancer survivors treated with TBI have more adipose tissue, less muscle mass, and worse muscle function compared to controls (41). Increased fat mass, in particular visceral adiposity, in TBI-treated survivors is associated with less insulin sensitivity (18). This “ectopic fat” profile, when observed along with marked hypertriglyceridemia, implicates adipocyte dysfunction and disordered fatty acid metabolism, reminiscent of lipodystrophy, in the pathogenesis of marked insulin resistance (42, 43).

The risk associated with abdominal radiation, used in the treatment of many solid tumors and certain lymphomas, is particularly increased in individuals with diagnosis and treatment at younger ages, older current age, higher BMI, and receipt of higher doses of radiation to the pancreatic tail (44). Radiation therapy to the pancreas leads to islet cell degranulation, mitochondrial destruction, and impaired insulin secretion (16). Human studies demonstrate smaller pancreatic volume on MRI after abdominal radiation, and a linear dose-dependent increased risk for developing DM with radiation to the tail of the pancreas (44–46). However, a recent study of two-year CCS with history of abdominal radiation found no difference in fasting insulin levels between those with normal vs. abnormal glucose tolerance (47). In contrast, in a study of young adult survivors of childhood ALL, those who received TBI as part HCT conditioning had a higher prevalence of abnormal glucose tolerance and lower pancreatic beta-cell reserve, less insulin secretion, and decreased pancreatic volume adjusted for body surface area, even years after TBI exposure compared to those with history of ALL who did not receive TBI and HCT (45). Together, these studies suggest that in the early survivorship period, pancreatic beta-cell damage, if present, is not clinically overt and does not account for the increased risk of DM in CCS. With time, the beta-cell damage can become more pronounced and plays a more important role in the pathogenesis of DM. Another contributary mechanism to developing DM after abdominal radiation is the effect of abdominal radiation on adipose tissue (48). Radiation is associated with decreased subcutaneous adipose tissue, which is thought to protect against metabolic dysregulation, and increased visceral adipose tissue, which increases metabolic dysregulation including insulin resistance (49, 50). Radiation-induced adipose tissue damage leads to inflammation and mitochondrial injury, both of which are also associated with the development of DM (51, 52). The extent to which radiation to the pancreas affects beta-cell function, and the timing of these effects relative to the radiation exposure deserve further delineation in future studies.

Cranial radiation is also associated with development of DM, though to a lesser degree than TBI and abdominal radiation (53). In a study of over 700 CCS with a history of cranial radiation, nearly 50% developed growth hormone deficiency (54). Untreated growth hormone deficiency is associated with alterations in body composition, including increased waist-to-height ratio and low muscle mass, that can contribute to insulin resistance (54). Cranial radiation may also impact hypothalamic responsiveness to leptin, leading to an increase in adipose tissue and its production of leptin (51). Impaired responsiveness to leptin would be expected to lead to increased caloric intake and subsequent weight gain, further increasing insulin resistance (55). Thus, both radiation exposure and increased adiposity can exacerbate similar tissue-specific inflammatory responses and mitochondrial dysfunction leading to insulin resistance. Hypothalamic-pituitary dysfunction with similar endocrine alterations as seen with cranial radiation, may also develop following neurosurgical tumor intervention (56).

### Chemotherapy

Corticosteroids, commonly used in the treatment of ALL and many lymphomas, are associated with increased risk of treatment-induced DM. The posited mechanisms include steroid-induced inhibition of glucose uptake in peripheral tissues, increased hepatic gluconeogenesis, and excess lipid deposition in skeletal muscle (51, 57, 58). While not all individuals who receive corticosteroids develop DM after completion of therapy, survivors who develop treatment-induced DM during steroid therapy are at increased risk of subsequently developing DM even when off steroids (59). There is also a well-described relationship between alkylator exposure and hypogonadism (60, 61). Hypogonadism can lead to alterations in body composition including excess adiposity, thereby increasing the risk of cardiometabolic disease (62). L-asparaginase increases the risk for developing DM in the acute treatment setting by decreasing insulin secretion in response to hyperglycemia (57, 58, 63, 64). However L-asparaginase is not associated with the development of DM following completion of treatment (16).

## Cellular processes implicated in aging- and treatment-related DM

DM has historically been considered an aging-related disease, and older age is an established risk factor for developing DM (14). In the general population, age-related changes, such as frailty (the most widely recognized phenotype of aging), mitochondrial dysfunction, and increased IMCL, can drive the development of DM (65, 66). CCS exhibit signs typically associated with premature or accelerated aging related to having received cancer treatments at a young age (67). At a mean age of 33 years, CCS demonstrate a prevalence of frailty similar to adults who are much older (≥65 years) (67). Compared to their siblings, CCS demonstrate a 6-fold higher cumulative incidence of severe or life-threatening chronic health conditions, such as hypertension, dyslipidemia, and DM, which are typically seen in older individuals (3, 68). Emerging research points to parallels in cellular processes implicated in aging- and cancer treatment-related DM. Mitochondrial dysfunction is a potential cellular mechanism underlying this accelerated aging phenotype, and studies show a decrease in mitochondrial content and respiration with aging (69–71). These alterations occur following exposure to common chemotherapeutic agents used in historical and contemporary regimens to treat pediatric cancers, such as alkylators and anthracyclines (58), or radiation therapy (72). For example, doxorubicin decreases skeletal muscle mitochondrial respiration and increases mitochondrial ROS production (73). The effects of radiation include impaired skeletal muscle fatty acid oxidation and mitochondrial oxidative capacity (72). Reduced mitochondrial quantity or function in individuals with DM has been observed in a number of studies (74–76). Specifically, individuals with DM have decreased respiration through the electron transport chain (75, 76). The decreased mitochondrial oxidative capacity may lead to lipid accumulation in skeletal muscle and thereby further impede insulin signaling (77). Thus, alterations of skeletal muscle metabolic processes due to cancer treatment are similar to the changes that occur in patients with diabetes. Additional investigations are needed to specifically elucidate the mechanisms underlying accelerated aging as they relate to the increased risk of DM in survivors of childhood cancer.

Genetic and gene-environment interactions also play an important role in the inter-individual variation in the risk of developing DM. Numerous germline genetic loci have been associated with DM both in oncology and non-oncology populations (78, 79). A recent study of CCS found an association between a novel germline genetic locus, rs55849673-A, that is associated with decreased expression of *ERCC6L2*, an excision repair protein involved in deoxyribonucleic acid (DNA) damage repair and mitochondrial function, and diabetes (78). The association between rs55949673 and DM was stronger in survivors not treated with abdominal radiation than in those treated with abdominal radiation. This highlights that, in the absence of abdominal radiation, genetic variations that alter mitochondrial function and response to DNA damage affect the risk of developing DM differentially in cancer survivors in the context of their treatment exposures than in the general population. It also suggests that the magnitude of DM risk attributable to radiation is far greater than the risk associated with previously identified genetic factors (78). Epigenetic processes are altered by environmental influences. Studies of epigenetic alterations like DNA methylation due to aging, changes in body composition, and stressors such as inflammation are also underway (80). Identification of such genetic and epigenetic risk factors across individuals with varied treatment exposures may help to better understand which individuals are at highest risk of developing comorbidities such as DM. Emerging high through sequencing technologies, genetic/epigenetic screening and personalized medicine can facilitate early detection for more rapid intervention to prevent progression. This has the potential to inform approaches to screening guidelines and development of interventions that are truly tailored to each individual’s unique risk factors for DM.

## Screening

Guidelines in the general population recommend testing for DM in any children, adolescents, or adults who are overweight or obese by BMI and have at least one risk factor, and in all adults at age 35 (81, 82). Fasting blood glucose, HbA1c, or oral glucose tolerance test are considered appropriate for screening. As described above, there are limitations to using BMI as a key criterion for DM screening, particularly in CCS. Fasting blood glucose and HbA1c can lead to over- or underestimation of IGT, and have known limitations in certain populations (83, 84). Additionally, the younger age of DM onset in CCS may warrant earlier survivor-specific screening.

The current Children’s Oncology Group Long-Term Follow-Up Guidelines recommend screening for impaired glucose metabolism with a fasting blood glucose or HbA1c every 2 years in those with history of TBI or abdominal radiation (85). The guidelines do not currently include specific screening for impaired glucose metabolism in those with history of cranial radiation, corticosteroid exposure, or neurosurgery that may affect the hypothalamic–pituitary axis. Additional studies and quantification of this risk in CCS will inform screening guidelines following these treatment exposures.

## Future directions

Advancements in survivorship research have highlighted the substantial burden of DM in the growing population of CCS, as well as the complexity of DM pathogenesis. DM is a markedly heterogeneous disease, and an emerging paradigm in the non-oncology population is the identification of groups or clusters of individuals with phenotypically distinct DM (e.g., insulin-resistant, insulin-deficient, age-related, and obesity-related) (86, 87). This has not been formally studied in CCS. However, the multimodal nature of the therapeutic protocols utilized to treat pediatric cancers additionally contributes to the substantial variation in clinical phenotype and further complicates efforts to understand the specific drivers and mechanisms underlying the development of DM. The current body of literature suggests a time-dependent combination of impaired insulin secretion and muscle and adipose tissue insulin resistance which may derive from changes in body composition, including increases in VAT, adipose dysfunction and skeletal muscle IMCL. A key question for future studies is how the mechanistic etiology of these changes differs among survivors, dependent on their individual characteristics and treatment exposures. Future studies are necessary to further delineate the knowledge gap regarding body composition profiles and their role in the development of DM in CCS compared to the general population, considering additional established risk factors, and to identify strategies to promote normal growth and development in CCS. Large-scale genomic studies are also needed in CCS to identify relevant inter-individual genetic variations among different racial and ethnic groups. Addressing these questions is critical to determine which survivors are at highest risk of developing DM. This information will aid in identifying phenotypically distinct DM subgroups specifically within the context of cancer survivors. Furthermore, it will help establish clinically relevant biomarkers, risk prediction models, and risk-based screening guidelines to identify individuals most vulnerable to developing DM. Lifestyle modifications and pharmacologic interventions may then be tailored to these different diabetic phenotypes, targeting specific underlying pathways (e.g., increasing skeletal muscle mass, hepatic lipid mobilization, decreasing inflammation) and optimal times during which to intervene. For example, certain patients may derive benefit from insulin secretagogues such as sulfonylureas and others from insulin sensitizers such as metformin and thiazolidinediones. Information learned from these initial observational and interventional mechanistic studies will form the foundation for larger trials of individualized approaches aimed at early treatment and prevention of DM in at-risk childhood cancer survivors.

## Conclusion

In summary, CCS have an increased burden of DM, an important contributor to additional morbidity and mortality, largely due to alterations resulting from their cancer treatment. Future studies aimed at characterizing the mechanisms underlying the development of DM, specifically in this patient population, will inform targeted prevention and treatment approaches that are necessary to improve the lives of cancer survivors.

## Author contributions

RB and SM-M: conceptualization and manuscript preparation. SA, SM, and RN: manuscript preparation. All authors contributed to the article and approved the submitted version.

## Funding

This work was supported, in part, by grants from the NIH [K12CA001727(RB), R01DK065073 (RN)] and Schaeffer Endowment Funds (RN). The content is solely the responsibility of the authors and does not necessarily represent the official views of the National Institutes of Health.

## Conflict of interest

The authors declare that the research was conducted in the absence of any commercial or financial relationships that could be construed as a potential conflict of interest.

The handling editor NS declared a past co-authorship with the author SA.

## Publisher’s note

All claims expressed in this article are solely those of the authors and do not necessarily represent those of their affiliated organizations, or those of the publisher, the editors and the reviewers. Any product that may be evaluated in this article, or claim that may be made by its manufacturer, is not guaranteed or endorsed by the publisher.
